# Multiple Amine-Contained POSS-Functionalized Organosilica Membranes for Gas Separation

**DOI:** 10.3390/membranes11030194

**Published:** 2021-03-11

**Authors:** Xiuxiu Ren, Masakoto Kanezashi, Meng Guo, Rong Xu, Jing Zhong, Toshinori Tsuru

**Affiliations:** 1 Jiangsu Key Laboratory of Advanced Catalytic Materials and Technology, School of Petrochemical Engineering, Changzhou University, Changzhou 213164, China; renxiuxiu@cczu.edu.cn (X.R.); guo@cczu.edu.cn (M.G.); xurong@cczu.edu.cn (R.X.); 2 Separation Engineering Laboratory, Department of Chemical Engineering, Hiroshima University, 1-4-1 Kagamiyama, Higashi-Hiroshima 739-8527, Japan; kanezashi@hiroshima-u.ac.jp

**Keywords:** organosilica membrane, amine group, POSS, nanocomposite, gas separation

## Abstract

A new polyhedral oligomeric silsesquioxane (POSS) designed with eight –(CH_2_)_3_–NH–(CH_2_)_2_–NH_2_ groups (PNEN) at its apexes was used as nanocomposite uploading into 1,2-bis(triethoxysilyl)ethane (BTESE)-derived organosilica to prepare mixed matrix membranes (MMMs) for gas separation. The mixtures of BTESE-PNEN were uniform with particle size of around 31 nm, which is larger than that of pure BTESE sols. The characterization of thermogravimetric (TG) and gas permeance indicates good thermal stability. A similar amine-contained material of 3-aminopropyltriethoxysilane (APTES) was doped into BTESE to prepare hybrid membranes through a copolymerized strategy as comparison. The pore size of the BTESE-PNEN membrane evaluated through a modified gas-translation model was larger than that of the BTESE-APTES hybrid membrane at the same concentration of additions, which resulted in different separation performance. The low values of E_p_(CO_2_)-E_p_(N_2_) and E_p_(N_2_) for the BTESE-PNEN membrane at a low concentration of PNEN were close to those of copolymerized BTESE-APTES-related hybrid membranes, which illustrates a potential CO_2_ separation performance by using a mixed matrix membrane strategy with multiple amine POSS as particles.

## 1. Introduction

Carbon dioxide as one of main greenhouse gases mostly generated from the combustion of fossil fuels is increasing every year, which has led to global climate and environmental problems [1]. Removal of CO_2_ from flue gas has been designed by membrane separation with an efficient process [2]. Among them, microporous organosilica membranes with good thermal and chemical stability have presented attractive applications in gas separation through polymeric preparation methods in recent years [3]. In addition, the microporous structures and chemical properties suitable for target molecular separation can be designed by functional organic groups in precursors [4,5,6].

As typical bridged organosilica membranes, 1,2-bis(triethoxysilyl)ethane (BTESE)-derived membranes have shown long stability in water steam as well as large permeance of H_2_ or CO_2_ in the range of 10^−5^–10^−8^ mol/(m^2^·s·Pa) for gas separation, which are potential membrane materials for industrial applications [7,8,9]. The organic groups of –CH_2_–CH_2_– in BTESE prohibited water contact with Si–O–Si groups with enhancement of hydrothermal stability, and also enlarged the pore size to some extent, to increase the gas permeance compared with pure inorganic silica membranes. However, the pore sizes of BTESE membranes reported in a range of 0.4–0.8 nm were excessively high [10], and were larger than the kinetic diameter of most gas molecules, such as He, H_2_, CO_2_, N_2_ or CH_4_, etc. The relatively large pore size resulted in moderate selectivities. In addition, the functional groups suitable for CO_2_ separation are limited in BTESE.

To facilitate CO_2_ transport, several papers investigated a strategy by copolymerization of amine-contained alkoxysilanes with BTESE to prepare hybrid organosilica membranes [11,12,13,14]. The pore size and affinity were tuned by the appropriate selection of material and composition. Xomeritakis et al. investigated 3-aminopropyl triethoxysilane (APTES) doped into tetraethoxysilane (TEOS)-derived silica membranes, which showed a higher selectivity of CO_2_/N_2_ over 88 with a relatively lower CO_2_ permeance around 10^−10^ mol/(m^2^·s·Pa) [15]. Yu et al. reported primary, secondary and tertiary amine materials respectively incorporated into BTESE membranes, which achieved CO_2_ permeance over 10^−8^ mol∙m^−2^∙s^−1^∙Pa^−1^ with moderate selectivity of CO_2_/N_2_ from 4 to 28 [16]. Guo et al. described APTES into bis(triethoxysilyl)acetylene (BTESA) with C≡C–derived hybrid membranes, which improved both CO_2_/N_2_ selectivity of 31–42 and CO_2_ permeance over 10^−7^ mol/(m^2^·s·Pa) [17]. The principles for these copolymerized organosilica membranes are complex and not clear.

Mixed matrix membranes (MMMs) as an emerging technology have experienced major expansion in gas separation applications, which can overcome the trade-off performance of polymeric membranes and solve a major issue in large scale production [18]. Due to much thinner layers of BTESE less than 200 nm, incorporation of suitable particles into BTESE to prepare MMMs is a rarely reported strategy [19]. Kong et al. prepared metal-organic framework (MOF) material doped into BTESE membranes, which showed improved selectivity of CO_2_/CH_4_ = 18.2 [20]. Compared with dozens of nanometers of MOFs, polyhedral oligomeric silsesquioxane (POSS) has a particle size of 1–3 nm, which may be more appropriate in BTESE thin layers. It has been reported as a filler in polymers for gas separation, which offers better dispersion and fewer interfacial gaps [21,22]. The rigid three-dimensional Si–O–Si cubic cage has eight organic groups at its apices to offer free volume and functional affinity. The amine groups in POSS also can be designed at its apices. Previously, we have reported a POSS with octabenzamidoproply (–PrNH–C=O–Ph) groups incorporated into BTESE membranes [23]. The mixed matrix BTESE-POSS membranes showed improved selectivity of H_2_/N_2_. Amine groups are well-known to be effective for facilitating CO_2_ transport [24]. In order to increase the contents of amine groups for CO_2_ separation, however, a large amount of POSS are added into BTESE in previous work [23], which may excessively occupy the pore volume of BTESE, resulting in lower CO_2_ permeance and related selectivity.

**Figure 1:**
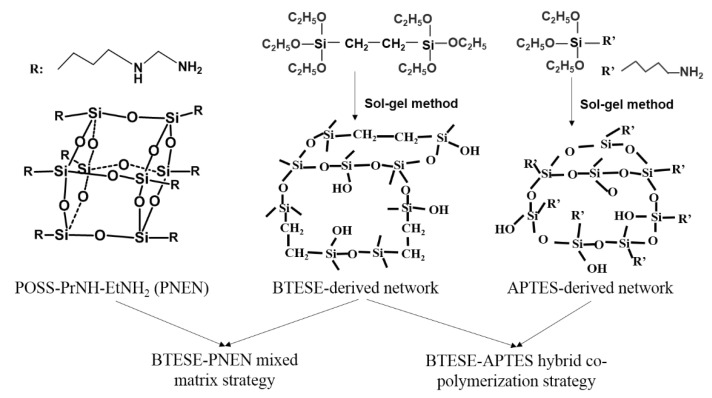
The schematic networks of 1,2-bis(triethoxysilyl)ethane (BTESE)-(–(CH_2_)_3_–NH–(CH_2_)_2_–NH_2_)(PNEN) and BTESE-3-aminopropyltriethoxysilane (APTES) hybrid sols.

To increase the amine content but with less content of POSS, in this work, we extend the concept to functionalize POSS nanoparticles containing multiple amine groups called Octa-N-(2-aminoethyl)-3-aminoproply-POSS (PNEN) into BTESE-derived sols to prepare mixed matrix membranes. It is composed of eight -PrNH-EtNH_2_ groups connected with the POSS cubic cage. A non-POSS additive using APTES with -PrNH_2_ groups connected with siloxane was used to fabricate BTESE-APTES hybrid membranes as comparison. The schematic networks of BTESE-PNEN mixed matrix and BTESE-APTES copolymerization used in this work by two strategies are shown in Figure 1. In what follows, gas separation performances of composite membranes were tested, and compared with organosilica membranes prepared by hybrid copolymerization strategy. These novel mixed matrix organosilica membranes may provide a comprehensive account of the influence of multiple amine functional group-containing POSS in BTESE for gas transport.

## 2. Materials and Methods 

### 2.1. Preparation of Sols

The organosilica sols were prepared via the hydrolysis-condensation method. First, BTESE was dissolved in ethanol, then water and acetic acid (HAc) as the catalyst were added under continuous stirring at 25 °C for 2 h. The molar ratio of the solution was BTESE:H_2_O:HAc:EtOH = 1:120:0.2:99. The pure Octa-N-(2-aminoethyl)-3-aminoproply-POSS (PNEN) kindly supplied by Nippon Shikubai Co. Ltd. was dissolved in ethanol at a concentration of 5 wt%. Then it was added into BTESE-derived sols in the molar ratio of 0.2 and stirred at 50 °C for 0–6 h. All mixed solutions were clear without any sediment or interfacial layers, exhibiting good compatibility between BTESE-derived sols and POSS in an ethanol solvent. Throughout this paper, the sols and the following membranes are named as BTESE or BTESE-PNEN according to the precursor used.

### 2.2. Preparation of Membranes

BTESE-PNEN composite membranes were fabricated on porous α-alumina tubes (porosity: 50%, outside diameter: 10 mm, average pore size: 1–2 μm) as supports. First, two types of α-Al_2_O_3_ particles (2 and 0.2 μm) were coated on the support and calcinated at 550 °C for 15 min. Then SiO_2_-ZrO_2_ sols were coated and also calcinated at 550 °C for 15 min. Finally, 0.5 wt% BTESE-PNEN solutions diluted by ethanol were deposited as the selective layer, followed by drying and calcination for 30 min under a N_2_ atmosphere. As a comparison, BTESE-APTES sols and membranes were prepared in a similar way as the steps of BTESE-PNEN sols and membranes.

### 2.3. Characterization of Sols and Membranes

The nanometer particle size of synthesized sols was determined by the dynamic light scattering zetazizer nano (ZEN3600, Malvern Co., Malvern, UK) instrument. The thermogravimetric mass spectrometer (TG-MS, TG-DTA-410S, Rigaku Co., Tokyo, Japan) was used for BTESE-PNEN powders with a heating rate of 10 °C/min under a He or He + O_2_ (He/O_2_ = 3, simulating air) stream to investigate thermal stability under an inert and oxidative atmosphere. The BTESE, PNEN and BTESE-PNEN films were prepared by spin-coating the respective sols with a concentration of 0.5 wt% on KBr followed by drying at room temperature or calcination at 300 °C under a N_2_ atmosphere for 30 min, and then were characterized by Fourier transform infrared (FT-IR) spectroscopy (FT-IR-4100, JASCO, Tokyo, Japan). The gas separation performances for these hybrid membranes were tested at a temperature ranging from 200 to 40 °C. The schematic diagram of the apparatus used for the gas permeation test was reported elsewhere [4]. These membranes were first dehydrated using N_2_ at 200 °C for 10 h on the feed side. An electric furnace was put outside of the membrane module to control the temperature. Then flow rate of a single gas of He, H_2_, CO_2_, CH_4_, CF_4_ or SF_6_ was tested by bubble film meter (Horiba Co. Ltd., Kyoto, Japan) in the permeate side of the membranes. The pressure drop was maintained at 1 bar between feed and permeate side, and the permeate stream was at atmospheric pressure.

## 3. Results

### 3.1. Characterization of BTESE-PNEN Powders and Films

**Figure 2:**
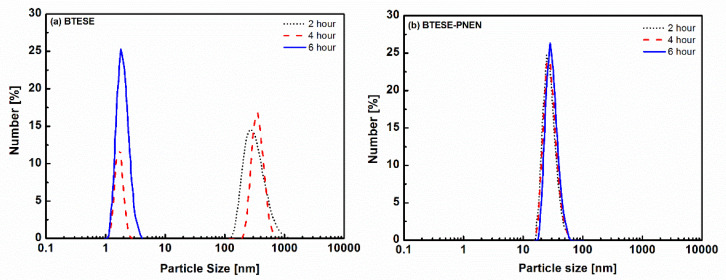
Particle sizes of (**a**) BTESE sols and (**b**) BTESE-PNEN sols (after reacting for 2 h) at different reaction times.

The particle sizes of BTESE and BTESE-PNEN sols as a function of reaction time are shown in Figure 2. The integrated networks of sols were formed by hydrolysis and polycondensation of the BTESE precursor by HAc as catalysis. The principles are as follows [25].

≡Si–OEt + H_2_O ⇔ ≡Si–OH + EtOH

≡Si–OH + ≡Si–OH ⇔ ≡Si–O–Si≡ + H_2_O

≡Si–OEt + ≡Si–OH ⇔ ≡Si–O–Si≡ + EtOH

The sol size of BTESE was larger than 100 nm after two hours and then decreased to around 2 nm after reaction for six hours. Most of the organosilica were catalyzed by a strong acid such as HCl or HNO_3_, and derived sols with similar particle sizes of several nanometers in less than two hours, which could form microporous structures [26,27]. In this work, the reaction rate for the weak acid of HAc is slower than that for strong acid. In order to obtain uniform and packed mixtures, PNEN dissolved in ethanol was added in two hour-reacted BTESE which is less hydrolyzed into sols. With a continuous stirring process, the particle size of mixtures was decreased to around 31 nm and was stable for six hours. The decreased size of BTESE may be due to the fast polycondensation reaction rate induced by catalytic amine groups in PNEN, which have been found in other research by using NH_3_∙HO_2_ or base-contained precursors [28,29].

**Figure 3:**
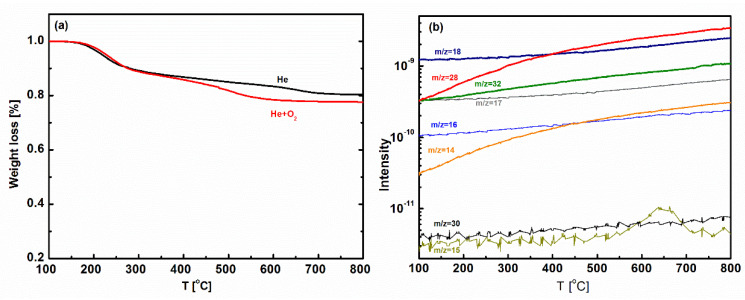
(**a**) Thermogravimetric (TG) curves of BTESE-PNEN powders under He and He+O_2_ atmospheres; (**b**) mass signals of BTESE-PNEN under He atmosphere.

The thermal stability of BTESE-PNEN is characterized by TG-MS measurement, as shown in Figure 3. The similar weight loss of BTESE-PNEN under He and He + O_2_ atmospheres were both smaller than 23% until 800 °C, indicating good thermal stability of materials under inert and oxidative atmospheres. The slight weight loss from 200 to 400 °C may be from the evaporation of water or ethyoxyl groups, which can be confirmed by TG-MS in Figure 3b. It can be found that the mass signal of m/z = 18 belonged to water and m/z = 14, 28 and 32 assigned to CH_2_–CH_2_ or CH_3_OH from the condensation of unreacted ethyoxyl or bridged groups in BTESE increased as temperature increased. Mass signals of m/z = 15, 16, 17, 30 are attributed to amine-containing groups (NH, NH_2_, NH_3_, CH_2_NH_2_) and did not increase obviously from 200 to 400 °C, indicating a stability of amine groups.

**Figure 4:**
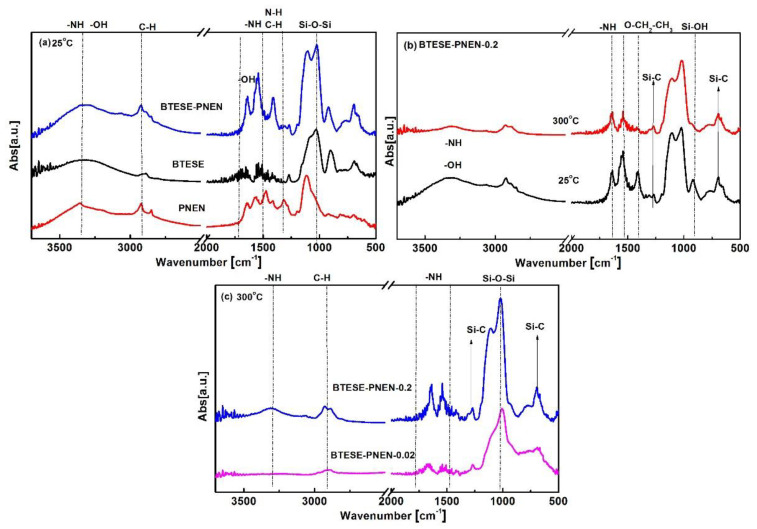
FT-IR spectra of (**a**) BTESE, PNEN and composite BTESE-PNEN (0.2) films calcination at 25 °C and (**b**) BTESE-PNEN-0.2 film calcination at 25 and 300 °C; (**c**) molar ratio of PNEN to BTESE at 0.2 and 0.02 formed films calcination at 300 °C in N_2_ atmosphere.

The FT-IR spectra for BTESE-PNEN films are presented in Figure 4. At 25 °C, the peaks for the BTESE-PNEN film are nearly the sum of BTESE and PNEN without any new peak appearance, indicating a physical mixture of BTESE and PNEN. The wavenumber of 1300, 1400, 1460 cm^−1^ is ascribed to C–H–N–H–related vibration for PNEN [30]. For BTESE, wavenumber from 1300 to 1500 cm^−1^ is ascribed to –CH_2_– or –CH_3_ vibration in Si–CH_2_– or –OEt [31]. The peaks at 1560 and 1640 cm^−1^ are ascribed to N–H groups in PNEN, and 1640 cm^−1^ is ascribed to OH groups in BTESE. From 1300 to 1640 cm^−1^, the peaks of C–H and N–H are overlapped, resulting in a combined three peaks for the BTESE-PNEN composites. As the temperature was increased from 25 to 300 °C, the characteristic peaks for the Si–OH groups at wavenumbers of 900 and 3300 cm^−1^ as well as –OEt at 1400 cm^−1^ disappeared, and simultaneously the peak intensity at 1000–1100 cm^−1^ assigned to Si–O–Si groups was increased, indicating that the hydrolyzed Si–OH and Si–O–Et groups in the sols could be further polymerized at high calcination temperature [32]. The characteristic peaks at 3300, 1650 and 1540 cm^−1^, attributed to N–H, and Si–C peaks at 1270–1290 cm^−1^ and 700 cm^−1^ [33], were kept well after 300 °C, indicating good thermal stability for the composite membranes. In addition, the peak intensity ascribed to NH– groups in PNEN was increased as the feed contents of PNEN increased (Figure 4c), indicating the PNEN contents in composite films can be tuned through the feed addition.

### 3.2. Gas Separation Performance for Composite Membranes

#### 3.2.1. Effect of Calcination Temperature

**Figure 5:**
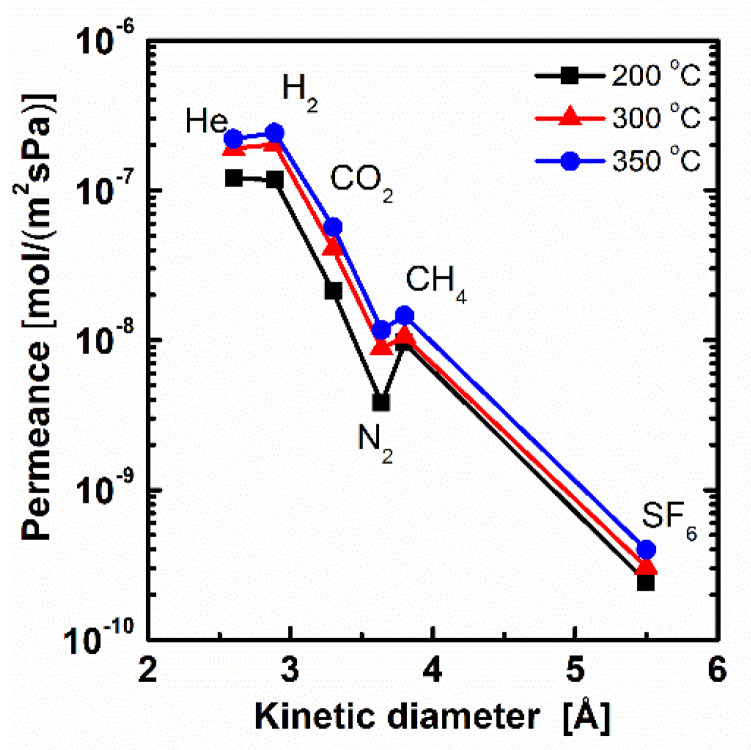
Gas permeance tested at 200 °C for BTESE-PNEN membrane calcinated at 200, 300 and 350 °C.

The calcination temperature is very important for membrane preparation by the sol-gel method. As the temperature is below 200 °C, the condensation degree of Si–OH in BTESE is too low to form integrated networks, which may result in lower selectivity [9,23]. As the temperature is larger than 350 °C, organic groups of –CH_2_–CH_2_– in BTESE may tend to decompose [34]. To keep the stability of organic groups and a certain condensation degree, composite membranes prepared by a molar ratio of PNEN to BTESE at 0.2 were chosen to calcinate at 200, 300 and 350 °C in N_2_ atmosphere. The gas permeation experiment was tested at 200 °C for comparison. Their gas permeance and permselectivity are presented in Figure 5 and Table 1. As the temperature elevated from 200 to 350 °C, the gas permeance of H_2_ was increased from 1.2 × 10^−7^ to 2.4 × 10^−7^ mol·m^−2^·s^−1^·Pa^−1^ (358–716 GPU), about twice the increases at high temperature. The permselectivities of H_2_/N_2_ were 30.9, 22.9 and 20.7 for membranes calcinated at 200, 300 and 350 °C, which were all higher than a Knudsen selectivity of 3.7, indicating defect-free membranes prepared with good thermal stability. In addition, the gas permeances for BTESE-PNEN membranes were sharply decreased as the kinetic diameter of gas increased, which indicated a molecular sieving mechanism.

The permselectivities of H_2_/N_2_ were lower at higher temperature. The effect of temperature on gas separation performance for this mixed matrix BTESE-PNEN membrane is different from other hybrid organosilica membranes. Qi et al. found a lower permeance of H_2_ with a higher permselectivity of H_2_/CO_2_ at a higher temperature by calcination of the Nb-doped BTESE membranes, which was explained as the denser network formed at higher temperature [34]. In this work, the network should also become denser due to condensation of Si–OH or Si–OEt at a higher temperature through FT-IR analysis. However, the PNEN particles with rigid POSS structure in BTESE sols may prohibit further condensation. Thus, some evaporated organic groups without condensation at high temperature generated large pores in BTESE-PNEN, which resulted in a larger gas permeance with lower permselectivity for small gas of H_2_/N_2_ and an increased permselectivity for large gas of H_2_/SF_6_. With respect to the high gas permeance and certain permselectivity, the BTESE-PNEN membranes calcinated at 300 °C were chosen and the membranes after were all calcinated at this temperature.

#### 3.2.2. Effect of PNEN Content

**Figure 6:**
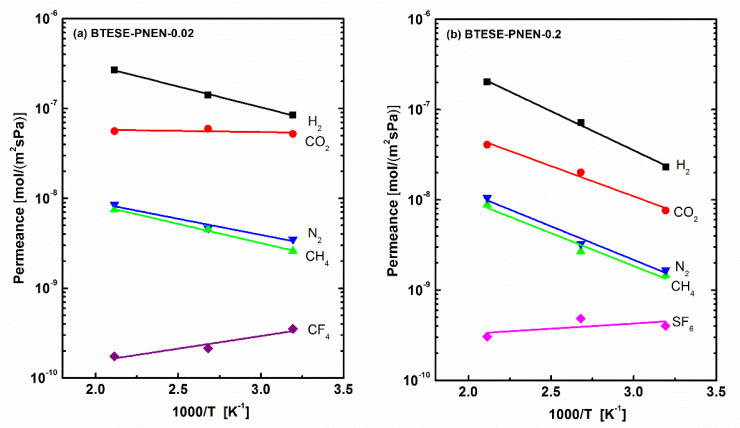
Temperature dependence of gas permeance of (**a**) BTESE-PNEN-0.02 and (**b**) BTESE-PNEN-0.2 mixed matrix membranes.

The larger POSS content reported previously in BTESE membranes caused the denser structures and reduced the gas permeance [23]. Thus, the low molar ratio of PNEN to BTESE of 0.02 was used to prepare membranes, and the single gas permeation property was compared with that of membranes above with the molar ratio of 0.2. The temperature dependence of gas permeance for the two membranes are shown in Figure 6. The corresponding gas permeance and related permselectivity of BTESE-PNEN-0.02 and 0.2 membranes tested at 200, 100 and 40 °C were shown in Table 2 and Table 3. The permeances of small gases (H_2_ and CO_2_) and their related permselectivities of H_2_/N_2_, H_2_/CH_4_ and CO_2_/N_2_ at all the temperatures were all higher for BTESE-PNEN-0.02 than those values for BTESE-PNEN-0.2 with high PNEN concentration. The separation performances of H_2_/N_2_ and H_2_/CH_4_ for BTESE-PNEN-0.02 were improved, compared with those of reported pure BTESE and related hybrid organosilica membranes [11,28]. For CO_2_ separation, BTESE-PNEN-0.02 membranes with multiple amine groups at a low concentration of 2% PNEN showed CO_2_ permeance in the range of 5–6 × 10^−8^ mol/(m^2^·s·Pa) (150–170 GPU) with permselectivity of CO_2_/N_2_ of 6.6–14.9, which was higher than values of BTESE-POSS (–PrNH–C=O–Ph) with single –NH groups [23]. The permselectivities of H_2_/CO_2_ were lower than the Knudsen selectivity of 4.7 both for BTESE-PNEN-0.2 and 0.02 membranes at low temperature, which also indicates favorable CO_2_ adsorptions on these multiple amine-contained organosilica membranes.

#### 3.2.3. Comparison of Non-POSS Material in BTESE Membranes

**Figure 7:**
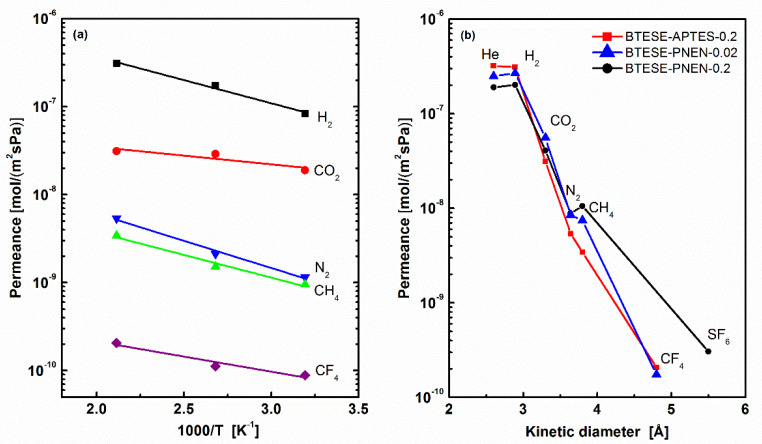
(**a**) Temperature dependence of gas permeance of BTESE-APTES-0.2 membrane and (**b**) kinetic diameter dependence of gas permeance of BTESE-APTES-0.2, BTESE-PNEN-0.2 and BTESE-PNEN-0.02 membranes at 200 °C.

Here, a non-POSS additive using APTES with a similar pendent of –PrNH_2_ groups connected with siloxane was used to fabricate BTESE-APTES membranes as comparison with BTESE-PNEN membranes for gas separation. The molar ratio of APTES to BTESE was kept at 0.2, and the other process was the same as BTESE-PNEN. The gas permeation of the BTESE-APTES-0.2 membrane was tested at 200, 100 to 40 °C, and its corresponding permeance and permselectivity as temperature dependence are shown in Figure 7a and Table 4. The comparison of gas permeance with BTESE-PNEN-0.2 and 0.02 tested at 200 °C is also shown in Figure 7b. All gases showed decreased permeance of BTESE-APTES-0.2 membrane with an increase of permeation temperature, indicating an activated diffusion of gases through this membrane. The permeances of small gases of He and H_2_ were higher than that of BTESE-PNEN-0.2 and 0.02, while the other gas was all lower, resulting in a larger permselectivity of H_2_/N_2_ and H_2_/CH_4_. The reduced H_2_/CO_2_ permselectivity at low temperature indicated the contribution of CO_2_ adsorptions on BTESE-APTES-0.2 membrane.

For three hybrid organosilica membranes, BTESE-PNEN-0.02, BTESE-PNEN-0.2 and BTESE-APTES-0.2, they showed a similar CO_2_ permeance in the range of 10^−8^–10^−7^ mol/(m^2^·s·Pa). The permselectivity of CO_2_/N_2_ is in the range of 5–16, a little higher than that of pure BTESE oraganosilica membranes reported with permselectivity of CO_2_/N_2_ 3–15 [12,27]. Compared with these organosilica membranes, the CO_2_/N_2_ separation performance of these amine-functionalized hybrid organosilica membranes did not improve too much.

## 4. Discussion

### 4.1. Estimation of Pore Size and Apparent Activation Energy

The gas permeation through microporous membranes usually obeys the solution-diffusivity mechanism. Thus, the pore size of membranes which determined the diffusivity of gas should be estimated. However, it is difficult to determine the pore size of silica membranes by conventional characterization methods, such as N_2_ physisorption-desorption or high-resolution electron microscopy. Considering the pore size is under nanometer range, a pore size determination with the temperature dependence of gas permeance *P_i_* based on the modified gas-translation (GT) model has been used as follows [10,35].

(1) $$P_{i}=\frac{\varepsilon_{i}}{3\tau_{i}L_{i}}\frac{{\left(d_{p}-d_{i}\right)}^{2}}{d_{p}^{2}}(d_{p}-d_{i})\sqrt{\frac{8}{\pi M_{i}RT}}\exp\left(-\frac{E_{P,i}}{RT}\right)=\frac{k_{0,i}}{\sqrt{M_{i}RT}}\exp\left(-\frac{E_{P,i}}{RT}\right)$$

where *k*_0,*i*_ is the pre-exponential factor defined by Equation (2), which can be expressed only by the configuration factors that include pore size, porosity, tortuosity and thickness are expressed as *d_p_*, $\varepsilon_{i}$, $\tau_{i}$ and *L_i_*, respectively. The parameter of *d_i_* is the kinetic diameter of gas *i*.

(2) $$k_{0,i}=\frac{\varepsilon_{i}}{3\tau_{i}L_{i}}\frac{{\left(d_{p}-d_{i}\right)}^{3}}{d_{p}^{2}}\sqrt{\frac{8}{\pi }}$$

The apparent activation energy of permeation *E_P,i_*, and the pre-exponential factor *k_0,i_*, can be obtained by regression using Equation (1) with the experimental temperature dependence of the single gas permeance data.

Based on the obtained *k*_0,*i*_, the mean effective pore size can also be determined using the following Equation (3):

(3) $$k_{0,i}=\frac{\varepsilon_{i}}{3\tau_{i}L_{i}}\frac{{\left(d_{p}-d_{i}\right)}^{3}}{d_{p}^{2}}\sqrt{\frac{8}{\pi }}=k_{0}{\left(d_{p}-d_{i}\right)}^{3}$$

Here, $k_{0}=\frac{\varepsilon_{i}}{3\tau_{i}L_{i}d_{p}^{2}}\sqrt{\frac{8}{\pi }}$. $k_{0}$ is a constant that depends only on the structure of the membrane pores and unrelated with the permeation molecules, and can be continued to be written as the cubic root way.

(4) $$\sqrt[{3}]{k_{0,i}}=\sqrt[{3}]{k_{0}}d_{p}-\sqrt[{3}]{k_{0}}d_{i}$$

Through the plot of $\sqrt[{3}]{k_{0,i}}$ against *d_i_* as a straight line, both the value of *d_p_* as an effective pore size for gas permeation and the structural parameter can be easily estimated.

### 4.2. Pore Size

**Figure 8:**
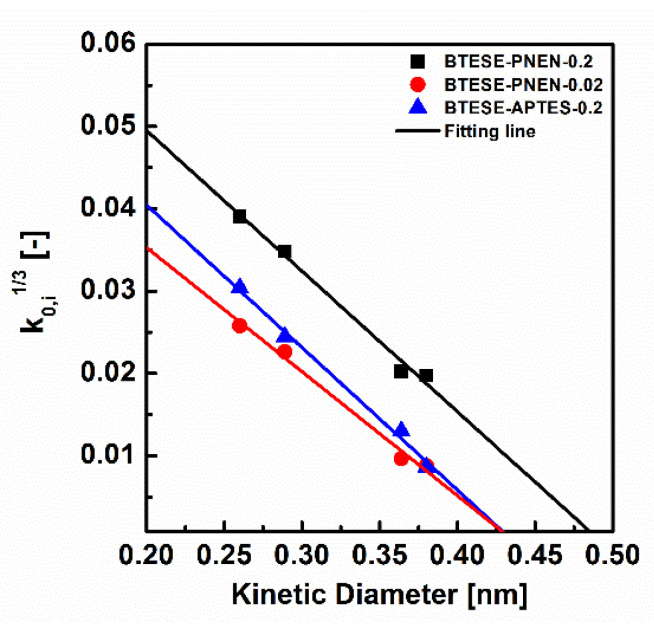
Relationship between kinetic diameters and *k_0,i_* for BTESE-PNEN-0.2, 0.02 and BTESE-APTES-0.2 membranes (symbols: experimental; solid curves: calculated based on Equation (4)).

Figure 8 shows the *k*_0,*i*_-related *d_i_* plot for membranes of BTESE-PNEN-0.2, BTESE-PNEN-0.02 and BTESE-APTES-0.2, and their pore size and apparent activation energy of E_p_ are summarized in Table 5. These functions are usually used to evaluate the pure organosilica membranes. For BTESE-PNEN and BTESE-APTES membranes, the fitting lines of R^2^ are all larger than 99%, which means the effective pore size determination method is also suitable for these mix matrix and hybrid membranes. The pore sizes of BTESE-PNEN-0.2, BTESE-PNEN-0.02 and BTESE-APTES-0.2 were 0.49, 0.43 and 0.43 nm, respectively. Most studies consider the pore size as the determination on gas selectivity [10,36]. The smaller pore size of BTESE-PNEN-0.02 reduced the transport of N_2_ or CH_4_ with relatively larger kinetic diameter of 0.364 nm or 0.38 nm than that of H_2_ (0.289 nm) and CO_2_ (0.33 nm), which can explain the larger selectivity of H_2_/N_2_, H_2_/CH_4_ and CO_2_/N_2_ than those values of the BTESE-PNEN-0.2 membrane. On the other hand, the BTESE-PNEN-0.02 membrane has the same pore size as BTESE-APTES-0.2, but presented lower permselectivity of H_2_/N_2_ or H_2_/CH_4_ than that of BTESE-APTES-0.2. From the fitting lines, we can find the slope of k_o_ is different for the two membranes. The larger slope of k_o_ for the BTESE-APTES-0.2 membrane probably was due to the higher porosity which was induced by the copolymerization of APTES and BTESE, than the physical mixing of POSS and BTESE. Thus, both the configuration factor and the effective pore size of membranes have effects on the gas separation performance.

### 4.3. Apparent Activation Energy

The apparent activation energy of gas permeation is a good indicator of the interaction between permeating molecules and the pore wall in a membrane. When the membrane became denser, activation energy was higher because of a larger repulsive force between gas and pore walls [37]. In Table 4, the order of activation energy of H_2_, CO_2_ and N_2_ for the mixed matrix membranes is: BTESE-PNEN-0.2 > BTESE-APTES-0.2 > BTESE-PNEN-0.02.

To analyze CO_2_ separation performance through these amine-containing membranes, the correlation between activation energy and their gas performance should be established. Based on the adsorption-diffusion mechanism for gas transport in microporous membranes, apparent activation energy E_p_ has a relationship with diffusion energy of *E_d_* and effective sorption enthalpy $\Delta H_{s}$ as follows.

(5) $$E_{p}=E_{d}+\Delta H_{s}$$

**Figure 9:**
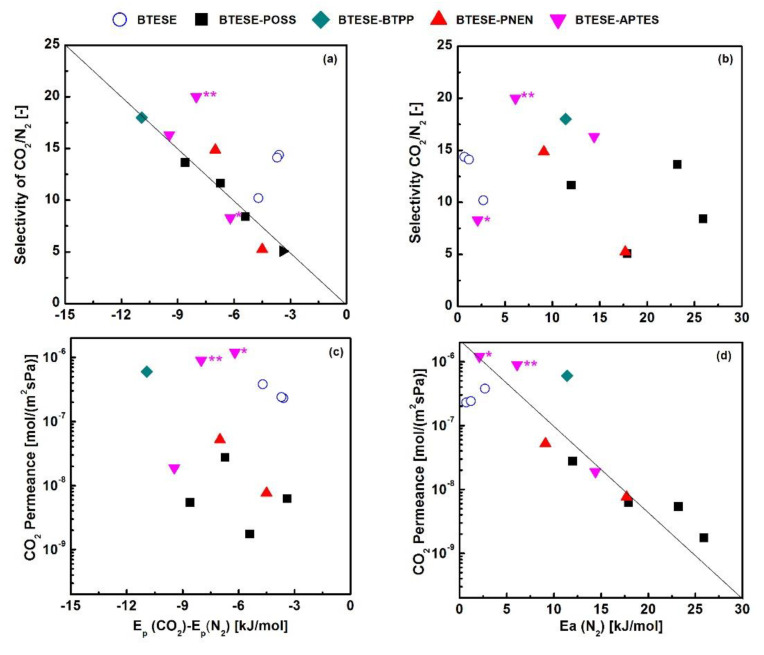
(**a**) Selectivity of CO_2_/N_2_ vs E_p_(CO_2_)-E_p_(N_2_) (**b**) selectivity of CO_2_/N_2_ vs E_p_(N_2_) (**c**) permeance of CO_2_ vs E_p_(CO_2_)-E_p_(N_2_) (**d**) permeance of CO_2_ vs E_p_(N_2_) for BTESE and related amine-hybrid organosilica membranes at 30–50 °C. (BTESE [40], BTESE-BTPP (50%) [38], BTESE-POSS (10–66.7%) [23], BTESE-PNEN (2, 20%) BTESE-APTES [this work], * BTESE-APTES (25%), ** BTESA-APTES (10%) [11,17]), BTPP: 4,6-bis(3-(triethoxysilyl)-1-propoxy)-1,3-pyrimidine; APTES: 3-aminopropyl triethoxysilane; POSS: octa-benzamidopropyl-POSS.

Considering the close kinetic diameter of CO_2_ (0.33 nm) and N_2_ (0.364 nm), the diffusivity energy of CO_2_ can be recognized equal to the E_p_(N_2_) (adsorption enthalpy of N_2_ is neglected). Thus, the adsorption enthalpy of CO_2_ can be described by E_p_(CO_2_)-E_p_(N_2_). Yu et al. used the relationship of E_p_(CO_2_) versus E_p_(CO_2_)-E_p_(N_2_) to discuss the CO_2_/N_2_ separation capability among hindered and unhindered amine-functionalized silica membranes, which showed greater potential in screening materials [15,17,28]. To roughly predict CO_2_/N_2_ separation performance, here, we also tried to separate the diffusivity and adsorption energy using E_p_(N_2_) and E_p_(CO_2_)-E_p_(N_2_) to analyze the properties of CO_2_ permeance and CO_2_/N_2_ selectivity, and compared with BTESE-related amine-containing hybrid organosilica membranes via copolymerization routes, as shown in Figure 9. It can be found that selectivity of CO_2_/N_2_ has a linear relationship with E_p_(CO_2_)-E_p_(N_2_) (Figure 9a), but it scattered as the function of E_p_(N_2_) (Figure 9b). This indicates that the adsorptions of CO_2_ on these amine-containing organosilica materials mostly depend on the separation ability. The lower energy of adsorption (E_p_(CO_2_)-E_p_(N_2_)), the higher the selectivity of CO_2_/N_2_. This is caused by interactions of CO_2_ with amine groups as well as higher solubility in an organic-rich phase [16]. The scattered points of CO_2_/N_2_ selectivity as the function of E_p_(N_2_) are ascribed to similar diffusion of CO_2_ and N_2_ with close kinetic diameter, and the sorption is ignored.

On the other hand, the permeance of CO_2_ is more closely linear to E_p_(N_2_) than that of CO_2_ adsorption energy as E_p_(CO_2_)-E_p_(N_2_) (Figure 9c,d), indicating that diffusion mainly determines the permeance of CO_2_. From Figure 7b, it can be found the gas permeation is mainly dominated by a molecular sieving mechanism in these microporous membranes. Thus, the diffusion (E_p_(N_2_)) rather than CO_2_ adsorption ability (E_p_(CO_2_)-E_p_(N_2_)) plays the roles in CO_2_ permeance. The linear relationship is established for both MMMs and hybrid membrane-contained amine groups. Compared with hybrid membranes such as BTESE-BTPP (50%), BTESE-APTES (25%) and BTESA-APTES (10%) which were prepared with high content of amine-containing groups [11,17,38], the BTESE-PNEN membranes can reach the close activation energy by using much less content of PNEN (2%) with multiple amine groups. The lower values of E_p_(CO_2_)-E_p_(N_2_) and E_p_(N_2_) for BTESE-PNEN-0.02 membranes illustrate a potential CO_2_ separation performance by using the MMMs strategy. By using Robeson’s upper bound correlations for comparison [39], it can be found that BTESE membranes modified by amine-containing groups in this work showed higher CO_2_ permeance of 1.9–5.2 × 10^−8^ mol/(m^2^·s·Pa) (28–78 Barrers, assuming the membrane thickness is 500 nm) and closer permselectivity of 14.9–16.3 than most polymeric membranes. In addition, BTESE-PNEN membranes showed a thermal stability even at 350 °C, which may be more favorable than polymers in some industrial applications.

## 5. Conclusions

Mixed matrix organosilica membranes BTESE-PNEN were prepared using multiple amine-containing POSS particles doped into BTESE-derived loose networks by the sol-gel method. The thermal stability of composite membranes was investigated by calcination membranes at 200, 300 and 350 °C. The gas separation performances were evaluated for membranes with molar ratio of PNEN to BTESE at 0.2 and 0.02. A similar amine precursor of APTES with molar ratio of 0.2 in BTESE by copolymerization strategy was used for comparison. Pore size distribution and apparent activation energy were determined by using a modified GT model through single gas permeation to analyze performance.

(1) The BTESE-PNEN mixed matrix membrane showed good thermal stability as the higher gas permselectivity of H_2_/larger molecules (N_2_, SF_6_ or CF_4_) than Knudsen values even after calcination above 350 °C.
(2) For CO_2_ permeance and permselectivity of CO_2_/N_2_, the order is: BTESE-PNEN-0.2 < BTESE-PNEN-0.02 ≈ BTESE-APTES-0.2.
(3) The pore sizes for BTESE-PNEN-0.2, BTESE-PNEN-0.02 and BTESE-APTES-0.2 were 0.49, 0.43 and 0.43 nm, respectively. Both the configuration factor and the effective pore size of membranes have effects on gas separation performance.
(4) A good linear correlation was presented for BTESE and related amine-containing organosilica membranes in CO_2_/N_2_ permselectivity versus E_p_(CO_2_)-E_p_(N_2_), or CO_2_ permeance versus E_p_(N_2_). The low values of E_p_(CO_2_)-E_p_(N_2_) and E_p_(N_2_) for BTESE-PNEN-0.02 membrane illustrates a potential CO_2_ separation performance by using MMMs strategy.

## Figures and Tables

**Table 1:** Gas permeance and permselectivity at different calcination temperatures.

Calcination Temperature/°C	H_2_ Permeance	Permselectivity			
	mol/(m^2^·s·Pa)	H_2_/N_2_	H_2_/CH_4_	H_2_/SF_6_	CO_2_/N_2_
200	1.2 × 10^−7^	30.9	12.2	481	5.6
300	2.0 × 10^−7^	22.9	19.1	659.8	4.6
350	2.4 × 10^−7^	20.7	16.6	604.2	4.9

**Table 2:** Gas permeance and related permselectivity of BTESE-PNEN-0.02 membrane.

Test Temperature/°C	H_2_ Permeance	CO_2_ Permeance	Permselectivity			
	mol/(m^2^·s·Pa)	mol/(m^2^·s·Pa)	H_2_/N_2_	H_2_/CH_4_	CO_2_/N_2_	H_2_/CO_2_
200	2.7 × 10^−7^	5.6 × 10^−8^	31.6	36.0	6.6	4.8
100	1.4 × 10^−7^	6.0 × 10^−8^	30.3	31.3	12.8	2.3
40	8.5 × 10^−8^	5.2 × 10^−8^	24.2	33.0	14.9	1.6

**Table 3:** Gas permeance and related permselectivity of BTESE-PNEN-0.2 membrane.

Test Temperature/°C	H_2_ Permeance	CO_2_ Permeance	Permselectivity			
	mol/(m^2^·s·Pa)	mol/(m^2^·s·Pa)	H_2_/N_2_	H_2_/CH_4_	CO_2_/N_2_	H_2_/CO_2_
200	2.0×10^−7^	4.1×10^−8^	22.9	19.1	4.6	4.9
100	7.1×10^−8^	2.0×10^−8^	26.4	21.9	7.5	3.6
40	2.3×10^−8^	7.6×10^−9^	15.9	13.9	5.2	3.0

**Table 4:** Gas permeance and related permselectivity of BTESE-APTES-0.2 membranes.

Test Temperature/°C	H_2_ Permeance	CO_2_ Permeance	Permselectivity			
	mol/(m^2^·s·Pa)	mol/(m^2^·s·Pa)	H_2_/N_2_	H_2_/CH_4_	CO_2_/N_2_	H_2_/CO_2_
200	3.1 × 10^−7^	3.1 × 10^−8^	57.6	90.5	5.8	10.0
100	1.7 × 10^−7^	2.9 × 10^−8^	81.5	114.3	13.7	5.9
40	8.3 × 10^−8^	1.9 × 10^−8^	71.6	87.5	16.3	4.4

**Table 5:** Pore size and apparent activation energy based on modified GT model.

Membrane	Fitting Plot	k_0_	Pore Size	E*p* (kJ/mol)		
	R^2^	-	nm	H_2_	N_2_	CO_2_
BTESE-PNEN-0.2	0.991	0.17	0.49	17.2	17.7	13.2
BTESE-PNEN-0.02	0.991	0.15	0.43	10.7	9.12	2.06
BTESE-APTES-0.2	0.994	0.17	0.43	11.1	14.4	4.95

## Data Availability

Not applicable.
